# Dental stewardship implementation and antimicrobial resistance awareness in India: prescribing patterns, knowledge gaps, and barriers–systematic review with narrative synthesis

**DOI:** 10.1017/ash.2026.10388

**Published:** 2026-05-18

**Authors:** Rasha Abdelsalam Elshenawy, Rishal Dsouza

**Affiliations:** Department of Medicine, School of Health, Medicine and Life Sciences, University of Hertfordshire, UK

## Abstract

**Introduction:**

Antimicrobial resistance (AMR) is a critical global health threat, with drug-resistant infections projected to cause up to 10 million deaths annually by 2050. India bears a disproportionate share of this burden. Dental practitioners contribute approximately 10% of national antibiotic prescriptions; however, antimicrobial stewardship (AMS) in dental settings remains underdeveloped, with no national dental-specific guidelines. This systematic review with narrative synthesis evaluates AMR awareness and AMS implementation among Indian dental professionals.

**Methods:**

A systematic review with narrative synthesis was conducted in accordance with PRISMA 2020 guidelines. Five databases were searched for studies published between January 2014 and December 2024. Eligible studies of any design examined AMR awareness, AMS behaviors, or antibiotic prescribing practices among Indian dental practitioners. Methodological quality was independently appraised using CASP checklists. Due to substantial heterogeneity across study designs and outcomes, findings were synthesized narratively and analyzed thematically.

**Results:**

From 1,852 records, 14 studies involving 3,602 participants were included. All studies referenced guidelines or clinical pathways. Prescribing practices were the most frequently assessed outcome (12/14; 86%), followed by compliance (9/14; 64%) and knowledge or awareness (6/14; 43%). Key findings included significant knowledge–practice gaps, inappropriate prescribing patterns, and the absence of structured stewardship infrastructure. Facilitators included baseline AMR awareness and professional willingness to engage, while barriers included curriculum deficiencies and limited access to guidelines.

**Conclusions:**

Structured dental AMS programs are both urgently needed and feasible in India. Addressing modifiable drivers through national prescribing guidelines, integrated AMS education, and audit and feedback systems is essential to support AMR containment.

## Graphical abstract

**Figure:**
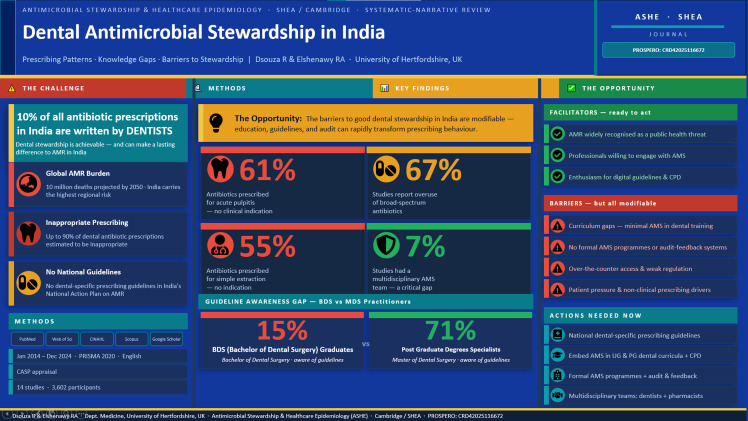

## Introduction

In his 1945 Nobel Prize Lecture, Sir Alexander Fleming warned that inadequate antibiotic dosing could enable bacteria to develop resistance. This insight has since materialized into one of the most serious global health challenges of the modern era.^1^ Today, antimicrobial resistance (AMR) undermines decades of medical progress, threatening to render common infections difficult to treat and routine procedures increasingly hazardous. The World Health Organization (WHO) identifies AMR as a major threat to global health, given its substantial contribution to mortality, morbidity, and economic loss worldwide.^2,3^ Projections from the O’Neill Review estimate that drug-resistant infections could cause 10 million deaths annually by 2,050.^4,5^

Antimicrobial stewardship (AMS)—coordinated, evidence-based interventions to optimize antibiotic choice, dose, route, and duration—has been strongly advocated by the UK Health Security Agency (UKHSA) and international bodies as a core strategy for appropriate antibiotic use across all healthcare settings.^6–8^

The WHO South-East Asia Region, which includes India, is recognized as having the highest burden and transmission risk of AMR globally.^9,10^ India, the world’s most populous country and one of the largest consumers of antibiotics, faces distinctive challenges, including high infection burdens, widespread over-the-counter antibiotic access, regulatory gaps, and significant variation in healthcare quality. Within this context, dentistry represents an important yet often overlooked sector.^11^ Dentists prescribe approximately 10% of all antibiotics,^12–14^ primarily for odontogenic infections, odontogenic cellulitis, and perioperative prophylaxis.^15,16^ Although antibiotics are essential for managing conditions such as dental abscesses or preventing infective endocarditis, they are intended to be adjuncts, not substitutes, for definitive dental treatment such as drainage, endodontic therapy, or extraction.^17,18^ Nevertheless, inappropriate dental prescribing, such as routine antibiotics for irreversible pulpitis, uncomplicated toothache, or simple extractions, remains widespread and contributes significantly to AMR.^19,20^

Despite the importance of rational prescribing, the undergraduate dental curriculum in India contains minimal content on antibiotic stewardship, resistance mechanisms, or evidence-based prescribing.^19^ A policy document analysis by Bhuvaraghan et al. confirmed the absence of dental-specific antibiotic prescribing guidelines within India’s key national policy documents, including the National Action Plan on AMR, and found no continuing professional development (CPD) materials directed at dental practitioners.^21^ Combined with a high burden of oral disease and consistently documented inappropriate prescribing practices,^22^ the need for structured dental AMS programs tailored to the Indian context is clear.

These challenges are not unique to India. Globally, dentists prescribe approximately 10% of all antibiotics, yet up to 90% of dental antibiotic prescriptions are estimated to be inappropriate.^23^ A systematic review of dentists’ knowledge, attitudes, and perceptions across 37 studies identified complacency, lack of guideline awareness, fear of adverse outcomes, and tendency to defer definitive treatment as the predominant drivers of inappropriate prescribing, all modifiable factors amenable to targeted intervention.^24^ In the United States, a prospective dental AMS intervention demonstrated that education combined with audit and personalized feedback improved appropriate antibiotic use from 19% to 87.9%, with a concurrent 14.5% reduction in total prescriptions.^25^ These findings highlights that structured AMS programs can rapidly and substantially optimize dental prescribing behavior across diverse healthcare settings.

Context-specific AMS interventions tailored to India’s epidemiological, regulatory, and socioeconomic environment are urgently needed.^19^

This systematic literature review aims to synthesize the current evidence regarding AMR awareness and AMS implementation among dental professionals in India. It identifies knowledge gaps, characterizes prescribing behavior, examines barriers and facilitators to effective stewardship, and proposes evidence-informed recommendations to strengthen AMS within Indian dental practice.

## Materials and methods

### Protocol registration and review framework

The protocol was prospectively registered (PROSPERO: CRD42025116672) and the review was conducted in accordance with PRISMA 2020 guidelines.

### Eligibility criteria

Eligibility criteria were established using the PICOS (Population, Intervention, Comparison, Outcome, Study design) framework to ensure systematic and comprehensive identification of relevant studies (Table 1).

**Table 1.** PICO framework for systematic review

PICO Framework	Description
**Population**	Dental professionals—students, interns, residents, general dentists, specialists, faculty and patients in dental settings where antibiotics are prescribed or used.
**Intervention**	AMS related exposure or intervention (e.g., guidelines, audits/feedback, education/CPD, decision support, policies, surveillance, prescribing restrictions), or studies assessing AMR/antibiotic use awareness/knowledge/attitudes/practices in dentistry.
**Comparison**	Standard practice/usual care, preintervention periods.
**Outcome**	Reviewing AMR and AMS in Indian Dentistry.
**Study design**	Randomized Controlled Trials (RCTs), cross-sectional studies, surveys, qualitative studies, case-control and cohort studies, narrative reviews, and interrupted time series designs.

PICO, population, intervention, comparison, outcome; AMS, antimicrobial stewardship; AMR, antimicrobial resistance; CPD, continuing professional development.

### Inclusion/exclusion criteria

Full eligibility criteria are detailed in Table 2. Studies examining AMR awareness, AMS practices, or antibiotic prescribing behaviors among Indian dental professionals (any design, English language, January 2014–December 2024) were included; systematic reviews, non-dental settings, and non-English publications were excluded.

**Table 2.** Inclusion and exclusion criteria for study selection

	Inclusion criteria	Exclusion criteria
**Participants**	Studies targeting the patient’s antibiotics use in dentistry. Dental professionals including students responsible for the prescription, dispensation, or administration of antibiotics	Studies not focused on dentistry patients and dentists administering antibiotics
**Intervention**	Studies that are focused on antimicrobial stewardship intervention	Studies not focused on antimicrobial stewardship intervention
**Comparison**	A comparison with control group in dentistry that dispensed antibiotics without antimicrobial stewardship intervention	–
**Outcome**	Reviewing the Antimicrobial resistance awareness. Reviewing the antimicrobial stewardship in India	–
**Study design**	Randomized Controlled Trials (RCTs), cross-sectional studies, surveys, qualitative studies, case-control and cohort studies, narrative reviews, and interrupted time series designs	Published systematic reviews and meta-analyses, case reports, case studies, and conference abstracts

### Information sources and search strategy

Five databases were searched (PubMed, Web of Science, CINAHL, Scopus, and Google Scholar) for studies published January 2014–December 2024, using Boolean combinations of terms related to AMR, stewardship, dentistry, and India, tailored to each database’s controlled vocabulary (Table 3).

**Table 3.** Electronic database search strategy

#	Search strategy
**1**	“Antimicrobial stewardship” OR “antibiotic stewardship” OR “antibiotic use” OR “antibiotic utilisation” OR “antibiotic utilization”
**2**	“dentistry” OR “dental practice” OR “dentist” OR “oral health” OR “dental professionals”
**3**	“awareness” OR “knowledge” OR “attitude” OR “perception”
**4**	“implementation” OR “intervention” OR “barrier” OR “facilitator” OR “adoption” OR “uptake”
**5**	“India”
**6**	1 AND 2 AND 3 AND 4
**7**	Limit to 2015 to 2025, Language: English

Databases Searched: PubMed, Web of Science, Cochrane Library, Scopus, and Google Scholar were systematically searched using the above strategy. The search was conducted in [August,2025] with final updates in [October, 2025].

### Study selection process

**Figure 1.**
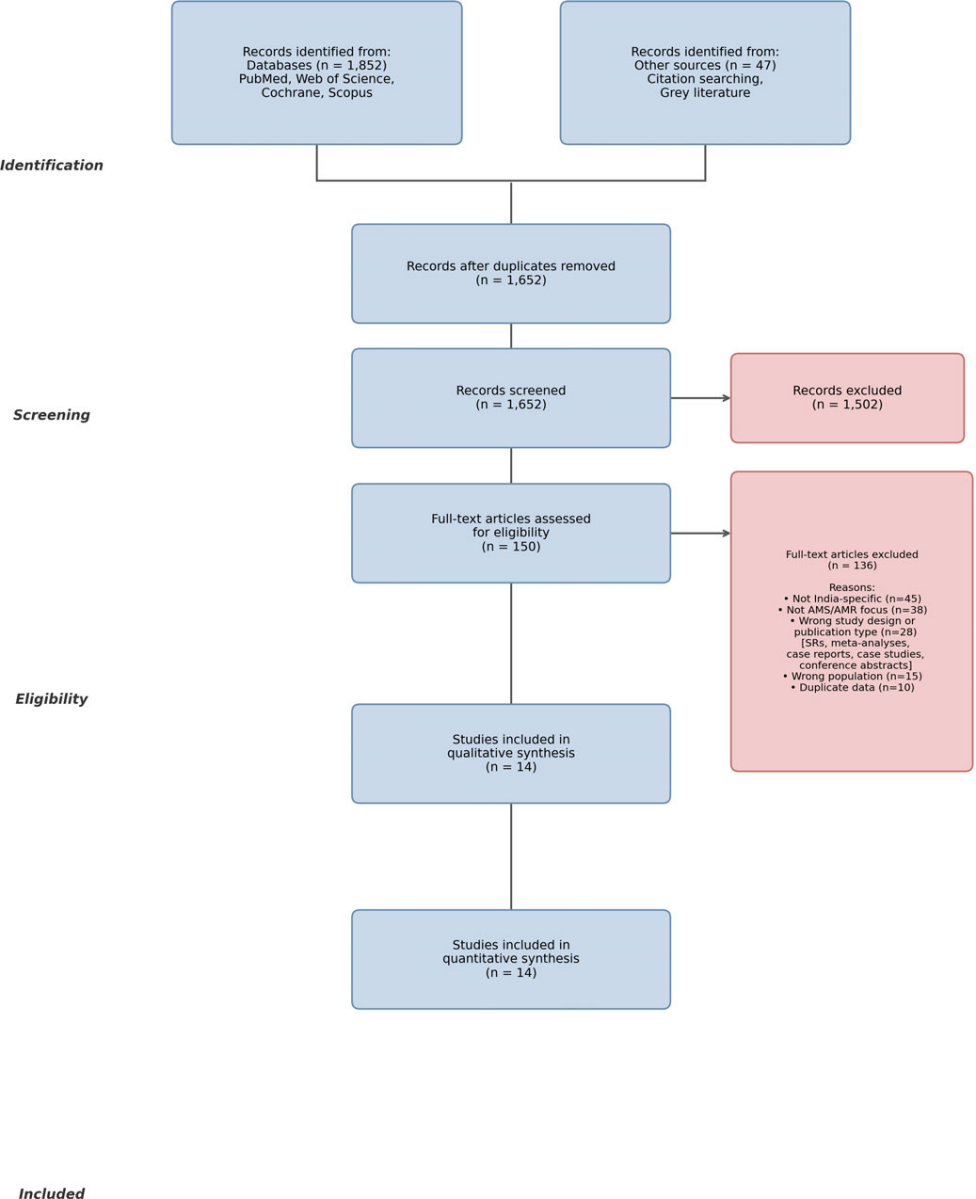
PRISMA 2020 flow diagram for systematic review of antimicrobial stewardship in Indian dentistry.

Following the comprehensive database search, all identified records were imported into Rayyan, an online systematic review management platform, and duplicates were removed. Titles and abstracts were independently screened by both authors (R.D. and R.A.E.) against the predetermined eligibility criteria, followed by full-text review of potentially relevant studies. Disagreements were resolved through discussion and consensus. The selection process is documented in the PRISMA flow diagram (Figure 1).

### Quality assessment

Regarding the quality assessment, both authors (R.A.E. and R.D.) independently appraised all included studies using the Critical Appraisal Skills Programme (CASP) checklists (CASP UK, 2018; available at casp-uk.net), selecting the appropriate checklist for each study’s design. Discrepancies were resolved through discussion and consensus. Detailed quality ratings for each included study are reported in Supplementary Table 1.

### Data extraction and synthesis

A standardized data extraction form captured study characteristics, participant details, AMR/AMS measures, prescribing behaviors, barriers and facilitators, and quality ratings. Data were extracted by one reviewer and verified by a second. Due to substantial heterogeneity, findings were narratively synthesized by grouping studies thematically and identifying consistent patterns.

## Results

### Study selection and characteristics

The search strategy identified 414 records, reduced to 357 after removing 38 duplicates and 19 non-eligible items. Title and abstract screening excluded 214 articles, leaving 143 full texts; 18 were inaccessible. A further 111 reports were excluded at full-text review for failing prespecified inclusion criteria: studies not addressing dentistry-related AMR/AMS outcomes (n = 45), absence of original empirical data such as editorials and opinion pieces (n = 38), wrong study design including published systematic reviews, meta-analyses, case reports, and conference abstracts (n = 28). Following the PRISMA process, 14 studies met all inclusion criteria and were included in the synthesis, representing 3,602 participants across Indian dental settings (Figure 1). The 14 included studies used the following designs: cross-sectional surveys, including Knowledge, Attitudes, and Practices surveys (11/14, 79%); narrative reviews (2/14, 14%); and one implementation evaluation (1/14, 7%) (Table 4).

### Antimicrobial resistance awareness and knowledge

Substantial AMR knowledge gaps were consistently reported. Most student-focused studies (4/6; 67%) identified insufficient understanding of AMR mechanisms and evidence-based prescribing. While AMR was widely recognized as a public health threat by practitioners (5/5; 100%), this did not equate to stewardship competence. Poor knowledge of WHO Good Prescribing practices, uncertainty about first-line antibiotic choices, and reliance on empirical experience rather than formal guidelines were common (Supplementary Table 1).

### Antibiotic prescribing practices and behaviors

**Figure 2.**
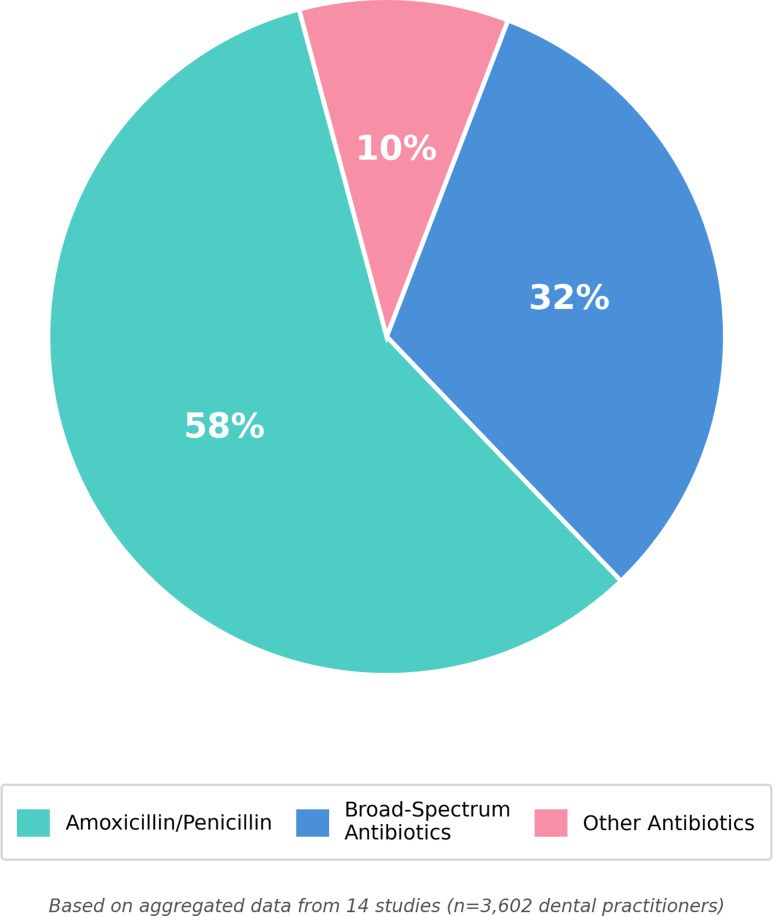
Antibiotic prescribing patterns in Indian dentistry.

Prescribing behaviors across the studies revealed high rates of inappropriate antibiotic use. Incorrect dosing and duration errors appeared in 5/14 studies (36%), while overuse of broad-spectrum antibiotics was reported in 8/14 studies (57%). The most commonly prescribed agents were narrow-spectrum penicillin-class antibiotics, particularly amoxicillin and penicillin V, which collectively accounted for 58% of documented prescriptions. Broad-spectrum antibiotics, including amoxicillin–clavulanate and other extended-spectrum agents, represented a further 32%, with remaining prescriptions (10%) comprising other antibiotic classes (Figure 2).

Antibiotics were prescribed for conditions manageable with local measures alone—including acute pulpitis, uncomplicated caries, and routine extractions—in 7/14 studies (50%), with additional reports of prophylactic use absent clear clinical indication (Supplementary Figure S1).

### Antimicrobial stewardship implementation and interventions

AMS activity within Indian dentistry was limited and fragmented. All 14 studies referenced guideline-based strategies, but only 1/14 (7%) incorporated a multidisciplinary AMS team and 1/14 (7%) implemented prospective audit and feedback. The most commonly assessed AMS-related outcomes were prescribing practices (12/14; 86%), compliance (8/14; 57%), and knowledge/awareness (6/14; 43%). Educational interventions in two studies showed short-term improvements in knowledge and prescribing intentions, though none included long-term validation (Table 5).

**Table 5.** AMS strategies and outcomes in implementing dental stewardship in India

Study ID/author(s) and year	AMS core strategies		AMS supplemental strategies	AMS outcomes		
	Multidisciplinary team	Prospective audit and feedback	Guidelines and Clinical pathways	Compliance	Prescribing practices	Knowledge/ awareness
(Chhabra et al., 2019)^26^	✓	X	✓	✓	✓	✓
(Doshi et al., 2017)^27^	X	X	X	X	✓	✓
(Jaber et al., 2024)^28^	X	X	✓	X	X	✓
(Kamate et al., 2023)^29^	X	X	✓	X	✓	X
(Lokhsadhan and Nasim, 2017)^30^	X	X	✓	✓	✓	X
(Manohar and Sharma, 2018)^31^	X	X	✓	X	✓	X
(Pung et al., 2016)^32^	X	X	✓	✓	✓	X
(Puranik et al., 2018)^33^	X	X	✓	X	✓	✓
(Ramachandran et al., 2019)^34^	X	X	✓	✓	✓	X
(Rela et al., 2021)^35^	X	X	✓	✓	✓	X
(Sharma et al., 2015)^36^	X	X	✓	X	X	✓
(Siddique et al., 2021)^37^	✓	X	✓	✓	✓	X
(Telang et al., 2021)^38^	X	✓	✓	✓	✓	X
(Vengidesh et al., 2023)^39^	X	X	✓	X	X	X
**Total (n = 14)**	**2**	**1**	**14**	**9**	**12**	**6**

Checkmarks (✓) indicate that the study reported the respective AMS strategy or outcome. AMS, antimicrobial stewardship. However, checkmarks (X) indicate that the study did not report the AMS strategies/outcomes.

### Facilitators and barriers to AMS

Thematic synthesis revealed both facilitators and barriers across the included studies. Facilitators identified included widespread recognition of AMR as a public health threat, professional willingness to engage with AMS activities, enthusiasm for digital guideline tools and continuous education, and leadership and institutional support where present. Educational barriers included curriculum deficiencies, a lack of CPD opportunities on AMR, and insufficient clinical training in evidence-based prescribing. Institutional barriers involved the absence of formal AMS programs, poor access to dental-specific guidelines, a lack of audit-feedback systems, and limited microbiology support. Regulatory challenges involved over-the-counter antibiotic availability, weak enforcement of prescribing restrictions, and minimal integration of dental prescribing data into AMR surveillance. Clinical barriers encompassed patient pressure, medico-legal concerns, and time constraints (Supplementary Figure S2).

### Qualitative thematic analysis

This was the most prevalent theme (12 of 14 studies). Across both student and practitioner groups, fundamental misunderstandings existed regarding antibiotic indications, resistance mechanisms, and dosing principles. Nearly half of resident doctors were unaware of WHO prescribing requirements,^26^ and guideline awareness was markedly lower among BDS (Bachelor of Dental Surgery) practitioners (15%) than MDS (Master of Dental Surgery) practitioners (71%).^34^ Several studies noted limited familiarity with prophylaxis standards, with only 67% of surgeons adhering to established recommendations.^40^

Despite high AMR awareness, inappropriate prescribing remained common for uncomplicated caries (53%), simple extraction (55%), and acute pulpitis (61%).^32,33,19^ Only 14% of practitioners were aware of national ADR systems,^33^ and non-clinical pressures including patient expectations and medico-legal concerns further drove overprescribing.^33,41^ BDS dentists showed higher overprescribing trends than MDS practitioners,^34^ and 57% were unable to identify alternatives for penicillin-allergic patients.^32^ Most studies (11/14) identified systemic weaknesses including inadequate stewardship coverage in curricula, absent formal AMS programs, and minimal audit mechanisms.^27,32,33^

## Discussion

This systematic review reveals a convergence of knowledge deficits, behavioral inconsistencies, and institutional barriers that collectively undermine rational antibiotic use in Indian dentistry.^26,33^ While AMR awareness was generally high, it did not reliably translate into appropriate prescribing, confirming that structural, educational, and behavioral reinforcement are needed alongside awareness.^32,34^

A central finding was the knowledge–practice gap: antibiotic prescribing for non-indicated conditions including dental caries (53%), simple extraction (54.5%), and acute pulpitis (60.9%) contradicted established guidelines.^17,27,29^ Non-clinical pressures—patient expectations, fear of litigation, and habitual prescribing patterns—drove this discordance over evidence-based decision-making.^32,34^

Guideline awareness varied markedly between BDS (15%) and MDS (71%) practitioners.^27,34^ Private practitioners relied heavily on empirical prescribing, reflecting limited institutional support,^29,32^ indicating that stewardship strategies must be tailored to practitioner background and workplace context.

These findings are not unique to India and reflect a global pattern of inadequate dental AMS. Vázquez-Cancela et al., in the most comprehensive systematic review of dentists’ knowledge, attitudes, and practices of antibiotic prescribing to date, examined 37 studies across multiple countries and found that inappropriate prescribing is predominantly driven by modifiable factors, including complacency, fear of litigation, lack of guideline trust, and tendency to defer definitive treatment, rather than by absence of awareness.^24^ This mirrors precisely the non-clinical pressures identified across our included Indian studies. In the United Kingdom, dentists account for approximately 10% of all primary care antibiotic prescriptions, and the UKHSA Dental Subgroup has acknowledged that prescribing rates have remained above prepandemic levels despite sustained stewardship efforts.^42^ Scotland’s Antimicrobial Prescribing Group has similarly identified the need for structured dental stewardship infrastructure, including audit tools, education programs, and CPD frameworks, to translate policy into practice.^43^

In the United States, Goff et al. demonstrated that combining targeted education with individualized audit and feedback improved appropriate antibiotic use among private practice dentists from 19% to 87.9%, yet this intervention reached only 15 dentists, underscoring the scale of the challenge in reaching the majority of practitioners who operate outside institutional oversight.^25^ The World Dental Federation has similarly called on national dental associations globally to embed AMS within national AMR action plans, recognizing that the dental profession carries a distinct and under-addressed responsibility in global resistance containment.^44^ Taken together, these international findings confirm that the knowledge–practice gap identified in Indian dentistry is a systemic global phenomenon, but one that is demonstrably amenable to structured, multifaceted intervention. The Indian context, characterized by a large prescriber population, absent national dental AMS guidelines, and minimal stewardship infrastructure, represents both an urgent priority and a significant opportunity for scalable impact.

Institutional and educational barriers are among the most entrenched challenges. Multiple studies identified the absence of formal AMS frameworks, limited guideline access, negligible ADR reporting, and insufficient continuing dental education (CDE) as key drivers of inappropriate prescribing.^26,33^ Nearly half of resident doctors were unaware of WHO prescribing guidelines, reflecting foundational undergraduate and postgraduate training gaps that structured curricula and CDE must address.^26,33,36^

These findings align with India’s National Action Plan on AMR priorities on education, training, and optimal antimicrobial use.^14,35^, Dentistry-specific guidelines and systems-level support—including decision-support tools, prescription monitoring, and audit feedback—are critical for translating policy into clinical practice.^33,40,37,38^

Taken together, these findings underscore the need for coordinated, multifaceted action across education, policy, and institutional frameworks to address the deep-rooted barriers to AMS implementation.

The findings of this review point to several clear and actionable recommendations. At the policy level, there is an urgent need to develop national dental-specific antibiotic prescribing guidelines and clinical pathways, as their absence represents one of the most consistently identified barriers across included studies. Prescribing practices must be formally measured as a core AMS outcome to monitor progress and drive improvement. At the institutional level, dental settings should establish formal AMS programs incorporating local guidelines, prescription monitoring, and adverse drug reaction reporting, with audit and feedback embedded as routine practice, since evidence consistently demonstrates that guidelines alone do not change prescribing behavior. At the educational level, undergraduate and postgraduate dental curricula must integrate practical AMS training, supported by CPD throughout practitioners’ careers. At the clinical level, individual dentists must strengthen diagnostic reasoning, consult guidelines regularly, and resist non-clinical pressures that drive unnecessary prescribing. Multidisciplinary collaboration involving pharmacists and microbiologists further enhances stewardship capacity. Meaningful and sustained improvements in dental antibiotic prescribing in India will require coordinated, aligned action across policy, education, institutions, and clinical practice. Future work should focus on implementation science to bridge the knowledge-practice gap. Priority areas include clinical decision-support tools, audit and feedback mechanisms, behavioral nudges, and digital guideline platforms. Evaluating these interventions across varied Indian practice settings will be critical to advancing effective dental stewardship.

This review has several important strengths. It provides a comprehensive synthesis of AMS implementation in Indian dental settings, addressing a critical evidence gap in a country with one of the highest global AMR burdens. The review followed rigorous PRISMA 2020 guidelines, employed dual independent screening with substantial inter-rater agreement, and applied CASP quality appraisal to all included studies. The synthesis draws on evidence from 3,602 dental professionals across diverse Indian practice settings, lending breadth and relevance to the findings. Collectively, the evidence provides a strong empirical basis for urgently developing India-specific dental AMS strategies that are tailored to the educational, regulatory, and institutional realities of the Indian dental context, rather than simply adopting frameworks designed for high-income healthcare systems.

Several limitations must be acknowledged. The restriction to English-language publications may have excluded relevant studies or gray literature published in Indian regional languages. The predominance of cross-sectional survey designs introduces susceptibility to social desirability bias, and the absence of longitudinal studies or randomized controlled trials limits conclusions about the sustained impact of AMS interventions on prescribing behavior. Substantial heterogeneity in study design, outcome measurement, and participant populations precluded meta-analysis and complicates direct comparisons across studies. The inclusion of two narrative reviews, while providing useful contextual insight, deviates from strict systematic review methodology. Finally, the findings may not be fully generalizable beyond the urban and dental college settings that predominated in the included studies, and the experiences of rural or private practitioners may be underrepresented.

## Conclusions

This review demonstrates that structured dental stewardship programs are urgently needed and achievable in India. While AMR awareness among Indian dental professionals is encouraging, translating this awareness into rational prescribing and effective stewardship practice remains the critical challenge. The findings of this study reveal that inappropriate antibiotic use is driven by modifiable factors including curriculum gaps, absent institutional frameworks, and non-clinical pressures, and strongly support the implementation of dental stewardship across Indian dental settings. Evidence from international dental stewardship programs further demonstrates that combining targeted education with audit and feedback can rapidly and substantially optimize antibiotic prescribing behavior. Developing national dental-specific prescribing guidelines, embedding AMS training within undergraduate and postgraduate education, and establishing formal audit and feedback mechanisms are practical, achievable, and necessary steps. Given that dentists account for approximately 10% of all antibiotic prescriptions in India, investing in dental stewardship can make a meaningful, lasting contribution to national and global efforts to combat AMR

**Table 4.** Characteristics of included studies assessing AMR awareness, antibiotic prescribing, and AMS practices among dental professionals in India

Study ID/Author(s) and year	Healthcare setting	Study design	Study population/participants	Sample size	Aims/objectives	AMR awareness	AMS practices/strategies/interventions	AMS outcomes
(Chhabra et al., 2019)^26^	Dental departments of 2 medical colleges	Quantitative survey	Junior resident dental doctors	70	Assess prescription knowledge, attitude, preference, and common errors	Gaps in knowledge; many unaware of WHO Good Prescribing	Education, guideline dissemination	Prescribing practices, compliance with prescription standards, knowledge scores
(Doshi et al., 2017)^27^	Dental colleges	Cross-sectional survey	Dental students	870	Evaluate knowledge and practices for antibiotics/analgesics	Variable awareness among students	Undergraduate curriculum exposure	Prescribing practices, awareness scores, appropriateness of antibiotic choice
(Jaber et al., 2024)^28^	Dental clinics (unspecified)	Cross sectional + lab	Dental professionals	304	Assess MRSA nasal carriage among dental professionals	NR	Infection control practices, awareness training	Resistance prevalence (MRSA carriage), awareness/knowledge scores
(Kamate et al., 2023)^29^	Dentistry context-India	Narrative review	NR	NR	Summarize AMR in dentistry and prevention strategies	Highlights awareness deficits and irrational prescribing	Education, rational prescribing, advocacy for stewardship	Prescribing practices (irrational vs rational use prevalence)
(Lokhasudhan and Nasim, 2017)^30^	Dental practitioners (South India)	Knowledge, attitudes and practices survey	Dental practitioners	315	Assess antibiotic use and attitudes	NR	CME, guideline reinforcement	Prescribing practices, self-reported compliance with stewardship guidelines
(Manohar and Sharma, 2018)^31^	General dentists and endodontists	Survey	Dental practitioners	150	Awareness of intracanal medicaments	NR	Structured education	Awareness scores, prescribing practices (choice of intracanal medicaments)
(Punj et al., 2018)^32^	Private dental clinics, Mangalore	Cross sectional survey	Private dental practitioners	173	Assess knowledge/awareness and prescription patterns	Awareness present but gaps on prophylaxis indications	Guideline use; stewardship policy	Prescribing practices (overuse/misuse frequency), compliance rates
(Puranik et al., 2018)^33^	Dental practices, Bangalore	Cross sectional survey	Dental practitioners (BDS/MDS)	400	Assess knowledge and practices; resistance awareness	Mixed awareness; indications inconsistent	Educational reinforcement, awareness campaigns	Knowledge/awareness scores, prescribing practices, inappropriate use prevalence
(Ramachandran et al., 2019)^34^	Multiple clinics (India)	Cross sectional survey	Dental practitioners (BDS/MDS)	361	Compare awareness and overprescription between BDS and MDS	General awareness present but many unaware of formal rules	Stewardship training, CME, guideline awareness	Prescribing practices, frequency of overprescription, compliance with rational use
(Rela et al., 2021)^40^	Urban dental practices, India	Cross sectional (questionnaire)	Dental surgeons (general dentists)	250	Inspect prescribing practices and knowledge of prophylaxis	Many follow guidelines: gaps remain	Partial guideline-based prophylaxis; standardized AMS protocols	Compliance rates with prophylaxis guidelines, prescribing practices
(Sharma et al., 2015)^36^	Medical and dental college	Questionnaire survey	Undergraduate medical and dental students	168	Assess attitudes toward AMS	Varied attitudes; need for AMS teaching	Curriculum inclusion, stewardship education	Awareness scores, attitudes toward AMS
(Siddique et al., 2021)^37^	Dental hospital/policy context	Narrative review	NA	NA	Outline challenges and opportunities for AMS in dentistry	NR	AMS framework (education, audit-feedback, stewardship teams)	Compliance rates and prescribing behavior
(Telang et al., 2021)^38^	Dental school (India)	Implementation evaluation	Students/faculty	231	Evaluate AMS improvements	NR	AMS program implemented (audit-feedback, education, surveillance)	Prescribing practices, compliance rates, improvements in rational use postintervention
(Vengidesh et al., 2023)^39^	Endodontic practice settings, India	Knowledge, attitudes and practices survey	Dentists involved in endodontic procedure	310	Assess prescription patterns for endodontic conditions	NR	Stewardship training, guideline adherence promoted	Prescribing practices, frequency of broad-spectrum antibiotic use, compliance rates

AMR, antimicrobial resistance; AMS, antimicrobial stewardship; BDS, bachelor of dental surgery; MDS, master of dental surgery; CME, continuing medical education; MRSA, methicillin-resistant Staphylococcus aureus; WHO, World Health Organization; NR, not reported. Sample sizes represent the total number of participants in each study.

## Supporting information

10.1017/ash.2026.10388.sm001 Abdelsalam Elshenawy and Dsouza supplementary material 1 Abdelsalam Elshenawy and Dsouza supplementary material

10.1017/ash.2026.10388.sm002 Abdelsalam Elshenawy and Dsouza supplementary material 2 Abdelsalam Elshenawy and Dsouza supplementary material

10.1017/ash.2026.10388.sm003 Abdelsalam Elshenawy and Dsouza supplementary material 3 Abdelsalam Elshenawy and Dsouza supplementary material

## Supplementary material

The supplementary material for this article can be found at https://doi.org/10.1017/ash.2026.10388.
