# Supplementary figures and images for: Dental stewardship implementation and antimicrobial resistance awareness in India: prescribing patterns, knowledge gaps, and barriers–systematic review with narrative synthesis
Source: Antimicrob Steward Healthc Epidemiol. 2026 May 18;6(1):e146. doi: 10.1017/ash.2026.10388 (PMC13184568; doi:10.1017/ash.2026.10388)

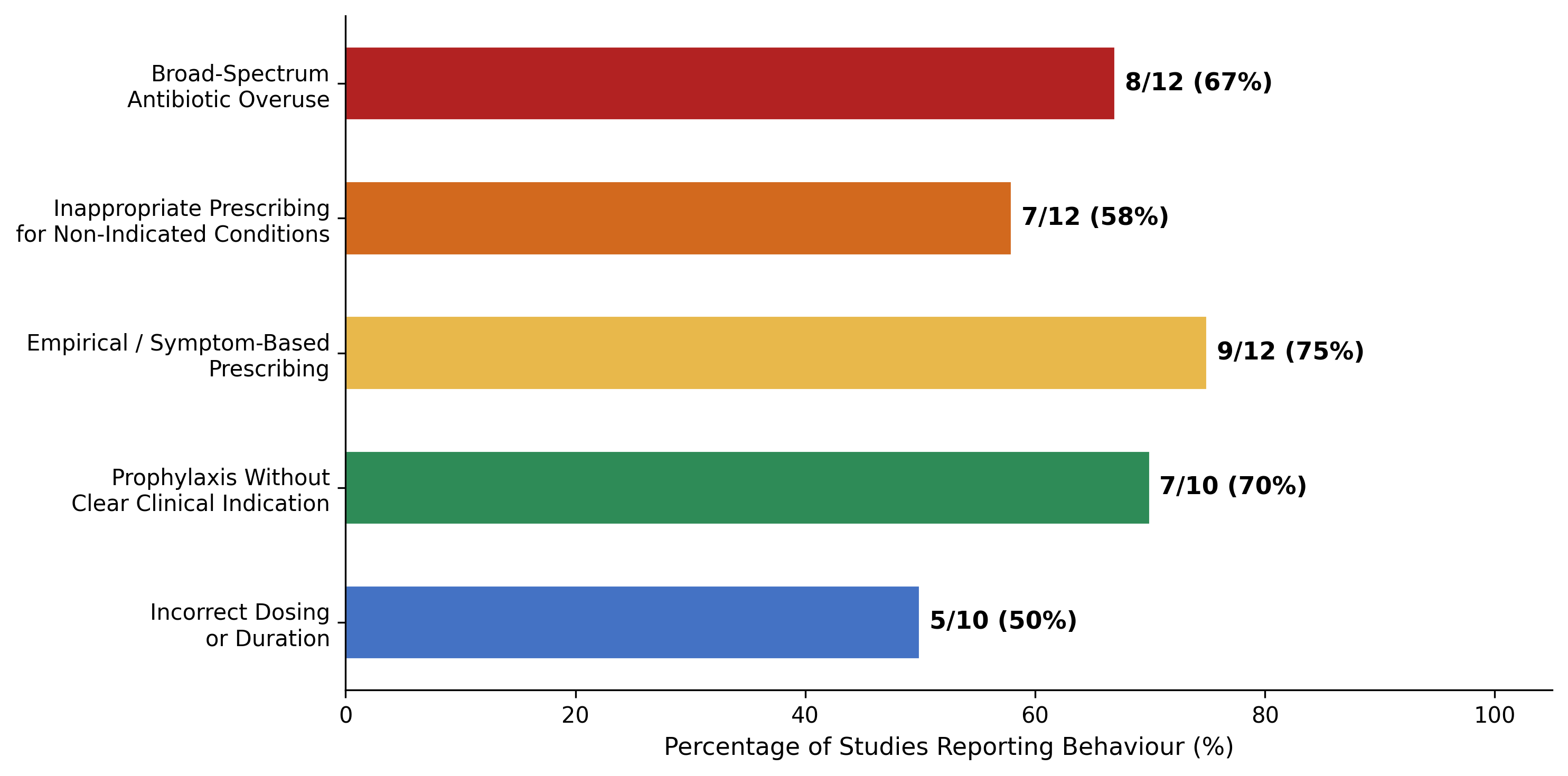

Supplement: Abdelsalam Elshenawy and Dsouza supplementary material 1 — Abdelsalam Elshenawy and Dsouza supplementary material [file S2732494X2610388Xsup001.tiff]

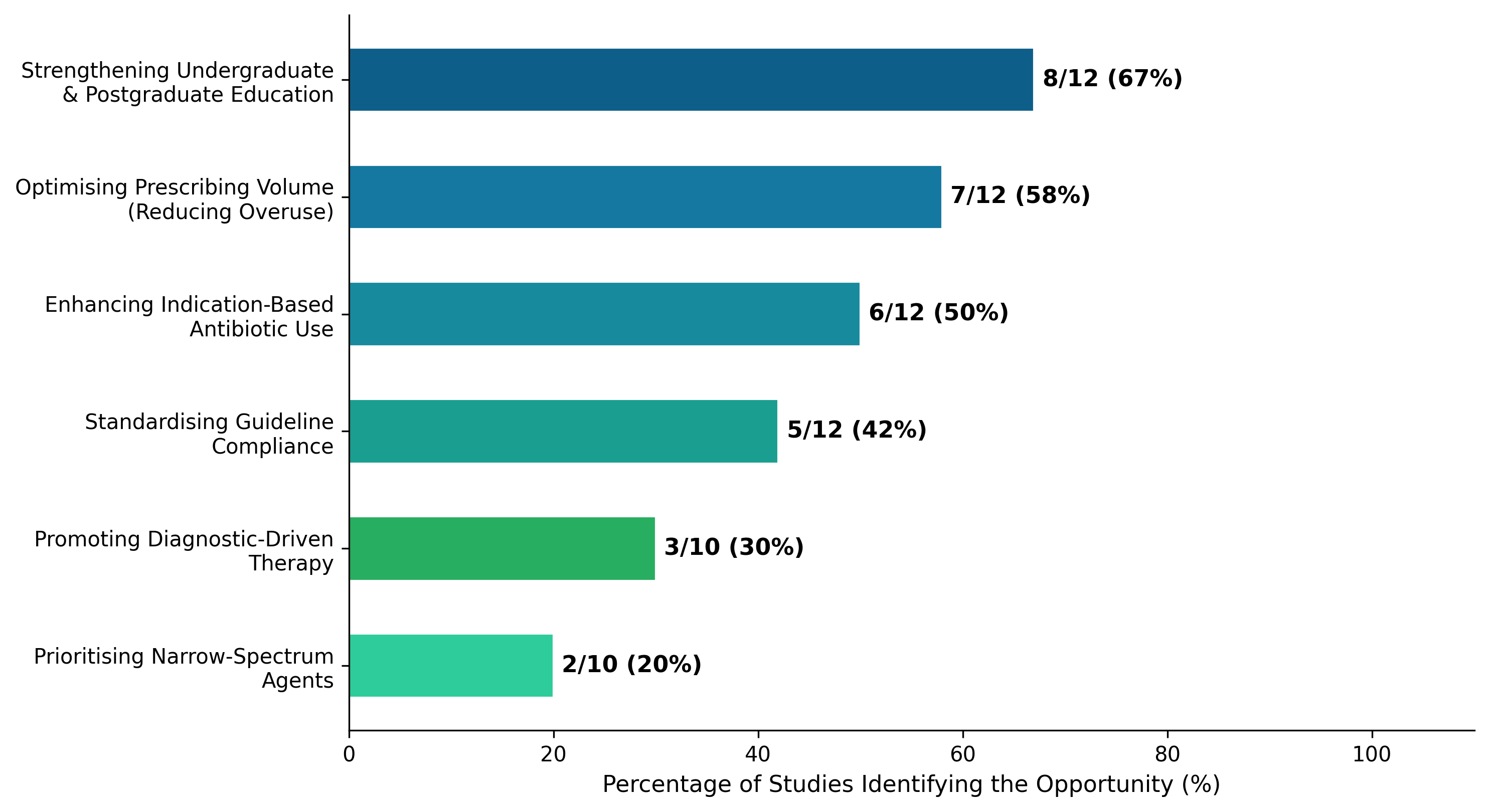

Supplement: Abdelsalam Elshenawy and Dsouza supplementary material 2 — Abdelsalam Elshenawy and Dsouza supplementary material [file S2732494X2610388Xsup002.tiff]
