# Supplementary material for: Dental stewardship implementation and antimicrobial resistance awareness in India: prescribing patterns, knowledge gaps, and barriers–systematic review with narrative synthesis
Source: Antimicrob Steward Healthc Epidemiol. 2026 May 18;6(1):e146. doi: 10.1017/ash.2026.10388 (PMC13184568; doi:10.1017/ash.2026.10388)
Supplement: Abdelsalam Elshenawy and Dsouza supplementary material 3 — Abdelsalam Elshenawy and Dsouza supplementary material [file S2732494X2610388Xsup003.docx]

**Supplementary Table 1.** Summary of Included Studies on AMR Awareness, Prescribing Practices, and Dental Stewardship Implementation in India

| **Study ID / Author(s) and Year** | **Facilitators of AMS implementation** | **Prescribing behaviour / compliance** | **Regulatory/ educational context** | **Key findings** | **Outcomes reported** | **Quality appraisal rating (CASP)** |
| --- | --- | --- | --- | --- | --- | --- |
| **Chhabra et al., 2019 (21)** | NR | Common errors included posology knowledge; reliance on faculty; amoxicillin widely prescribed | Undergraduate curriculum context | Resident dentists showed prescribing errors and insufficient AMS training. | Knowledge gaps and frequent errors; need for improved education | **Low** |
| **Doshi et al., 2017(22)** | NR | Reported prescribing intentions varied; need for curriculum strengthening | NR | Dental students had patchy antibiotic knowledge and poor guideline adherence. | Suboptimal knowledge in areas; training gaps highlighted | **Moderate** |
| **Jaber et al., 2024(23)** | NR | NR | NR | MRSA carriage was detected among dentists with only moderate awareness. | NR | **Moderate** |
| **Kamate et al., 2023(24)** | Guidelines, education, leadership emphasized | NR | NR | Narrative review highlighted irrational dental prescribing and called for AMS guidelines. | Proposes flowchart and strategies to reduce AMR in dentistry | **Low** |
| **Lokhasudhan & Nasim, 2017(25)** | NR | NR | NR | Practitioners had mixed knowledge and frequent empirical prescribing. | NR | **Moderate** |
| **Manohar & Sharma, 2018(26)** | NR | NR | NR | Dentists varied in awareness of intracanal medicaments, showing training gaps. | NR | **Moderate** |
| **Punj et al., 2018(27)** | NR | High daily prescribing; penicillin/amoxicillin first-line; frequent postop prophylaxis | Calls for guidelines and CME to standardize prescribing | Private dentists often prescribed antibiotics beyond evidence-based need. | Therapeutic prescribing varied and often suboptimal | **Moderate** |
| **Puranik et al., 2018(28)** | NR | Antibiotics often for routine conditions; 5-day courses common | NR | Resistance knowledge existed but did not consistently shape practice. | Knowledge-practice gaps; need for stewardship education | **Moderate** |
| **Ramachandran et al., 2019(29)** | NR | Overprescription higher in BDS; amoxicillin most common; differing durations (3 vs 5 days) | NR | Overprescription was widespread and influenced by clinician qualification | Highlights overuse and need for guideline adherence | **Moderate** |
| **Rela et al., 2021(30)** | NR | 67.6% followed formal guidelines; variability in choices for penicillin allergy | Reference to AHA/AAOS guidelines | Urban dentists showed partial adherence to prophylaxis guidelines with variability. | Inconsistent adoption of prophylaxis guidance | **Moderate** |
| **Sharma & sharma, 2015(31)** | NR | NR | NR | Undergraduates supported stewardship but lacked structured training. | NR | **Low** |
| **Siddique et al., 2021(32)** | Leadership and policy support as enablers | NR | NR | AMS framework proposed with key institutional barriers and facilitators. | Framework for implementing AMS in dental settings | **Low** |
| **Telang et al., 2021(33)** | NR | NR | NR | Dental school AMS programme improved antibiotic prescribing practices. | NR | **Moderate** |
| **Vengidesh et al., 2023(34)** | NR | NR | NR | Endodontic practitioners showed overuse of broad-spectrum antibiotics. | NR | **Moderate** |

*AMR = Antimicrobial Resistance; AMS = Antimicrobial Stewardship; BDS = Bachelor of Dental Surgery; CASP = Critical Appraisal Skills Programme; CME = Continuing Medical Education; MRSA = Methicillin-Resistant Staphylococcus aureus; AHA = American Heart Association; AAOS = American Academy of Orthopaedic Surgeons. Quality ratings based on CASP checklist: color-coded cells indicate Low (pink), Moderate (yellow), and High (green) quality ratings.*

**Supplementary Figure S1.** Problematic Prescribing Behaviours in Indian Dental Settings

*Abbreviations: NR = Not Reported (data not described by study authors); KAP = Knowledge, Attitudes, and Practices; CASP = Critical Appraisal Skills Programme.*

*This figure shows the frequency with which five specific problematic prescribing behaviours were reported across the included studies in Indian dental settings. Bars represent the number of studies reporting each behaviour as a proportion of the number of studies that specifically assessed that behaviour (feature-specific denominator), rather than the total of 14 included studies. This approach provides a more accurate estimate of the prevalence of each behaviour within the subset of studies designed to capture it.*

*Source: Narrative synthesis of 14 included studies representing 3,602 dental professionals across Indian dental settings, 2014–2024. Multiple behaviours may be reported within a single study.*

**Supplementary Figure S2.** Improvement Opportunities in Antibiotic Prescribing Practices to Promote Implementation of Dental Stewardship

*This figure presents six key improvement opportunities in antibiotic prescribing practices identified from the systematic narrative synthesis of 14 included studies on dental antimicrobial stewardship in India. Bars represent the number of studies identifying each opportunity as a proportion of the number of studies that specifically addressed the relevant improvement domain (feature-specific denominator), rather than the total of 14 included studies.*


Abbreviations: NR = Not Reported (data not described by original study authors); CPD = Continuing Professional Development; AMS = Antimicrobial Stewardship; WHO = World Health Organization; AHA = American Heart Association; AAOS = American Academy of Orthopaedic Surgeons.

Source: Narrative synthesis of 14 included studies representing 3,602 dental professionals across Indian dental settings, 2014–2024. Studies may identify multiple improvement opportunities.
